# Preoperative Patellofemoral Malalignment Worsened the Outcome of Patients after Total Knee Arthroplasty

**DOI:** 10.1055/a-2618-4666

**Published:** 2025-06-10

**Authors:** Nianlai Huang, Liangming Wang, Liquan Cai, Qingfeng Ke, Shiqiang Wu

**Affiliations:** 1 Department of Orthopedic, The Second Affiliated Hospital of Fujian Medical University, Quanzhou, China

**Keywords:** patellofemoral malalignment, patellofemoral alignment, knee osteoarthritis, total knee arthroplasty

## Abstract

The impact of preoperative patellofemoral malalignment (PFM) on the prognosis of patients who underwent total knee arthroplasty (TKA) remains unknown. This study aimed to explore the effect of preoperative PFM on the prognosis of patients who underwent TKA. This retrospective observational study included patients who underwent TKA at the Second Hospital Affiliated to Fujian Medical University between February 2018 and July 2020. The primary outcome measure was the Hospital for Special Surgery Knee-Rating Scale (HSS) score. The secondary outcomes included postoperative radiographic parameters (X-rays) and the occurrence of complications. A total of 94 patients (107 knees) who underwent TKA were included in the study. Of these, 37 knees had PFM and 70 had normal patellofemoral alignment (PFA). Patients with preoperative PFM showed a change in patellar outward displacement from 7.01 ± 3.91 mm preoperatively to −0.31 ± 2.86 mm postoperatively ( *p* < 0.001), and the lateral patellar tilt angle changed from 9.45 ± 7.47 degrees to 6.06 ± 3.61 degrees ( *P* = 0.009). Postoperative radiographic parameters between the PFM and PFA groups did not show any significant difference ( *p* > 0.05), but the postoperative HSS score in the PFM group was lower than in the PFA group (total score: 70.35 ± 8.39 vs. 80.47 ± 5.44, *p* < 0.001). In addition, 13 (35.14%) knees in the PFM group experienced postoperative anterior knee pain compared to 10 (14.29%) knees in the PFA group ( *P* = 0.013). Preoperative PFM may have an impact on the HSS score and the occurrence of anterior knee pain in patients after TKA. These findings suggest that surgeons should carefully evaluate preoperative PFA in patients undergoing TKA. Furthermore, patients with PFM may require additional monitoring and management of postoperative anterior knee pain, as well as special considerations for optimizing functional outcomes.

One common cause of postoperative failure after total knee arthroplasty (TKA) is patellofemoral complications, which can affect up to 8% of TKA patients. This can lead to poor prognosis and ultimately treatment failure. 1 Furthermore, patellofemoral complications are a major cause of surgical revision after TKA. 2 The most common cause of these complications is patellofemoral malalignment (PFM), which can result in issues such as patellar wear, osteonecrosis, fractures, subluxation, and dislocation. 3 4 Patellar trajectory refers to the dynamic relationship between the patella and the trochlea (groove in the thigh bone) during knee joint movement. 5 Imbalances in the patellofemoral trajectory, known as PFM, is often caused by anatomical morphological abnormalities and is common in younger individuals, especially young women. 5 The main function of the patellofemoral joint is to strengthen the quadriceps muscle, which helps strengthen the knee. 6 Excessive soft-tissue tension can lead to poor patellofemoral trajectory and decreased stability in knee. 7 By improving the balance of the soft tissues, complications such as anterior knee pain, patellar subluxation, and excessive wear and loosening of the patellar components can be reduced. 8 9 A study by Narkbunnam et al 10 11 found a strong connection between postoperative patellofemoral alignment and poor clinical outcome scores in patients who underwent primary TKA. However, there are few studies that specifically examined the impact of preoperative PFM on the recovery and prognosis of patients with TKA.

This study aimed to investigate the influence of preoperative PFM on the prognosis of patients who have undergone TKA. This study hypothesizes that there is a correlation between postoperative knee anterior pain and poor patellofemoral joint trajectory after TKA, and patients with poor patellofemoral joint trajectory are expected to exhibit lower postoperative HSS scores compared to patients with normal patellofemoral joint trajectory. To verify the hypothesis, we examined the impact of preoperative PFM on the outcomes of TKA.

## Methods

### Study Design and Participants

This retrospective observational study analyzed patients who underwent TKA at the Second Hospital Affiliated to Fujian Medical University between February 2018 and July 2020. The inclusion criteria of patients were: (1) patients diagnosed with primary knee osteoarthritis (KOA) based on the International Federation of Patellofemoral Osteoarthritis Expert Consensus (2017 version) 12 ; (2) patients with complete medical record; and (3) patients with complete imaging data of axial X-ray with the knee flexing at 15 degrees before and after the operation. The exclusion criteria of patients were: (1) osteoarthritis and bone deformities due to trauma; (2) replaced patella; (3) patients who underwent previous knee joint surgery prior to TKA; or (4) patients who developed post-TKA infection. Patients with lateral patellar displacement >5 mm or a patellar tilt angle >15 degrees were included in the PFM group, while the remaining patients were categorized into the normal patellofemoral alignment (PFA) group.

### Procedures

A total of 230 patients (253 knees) who underwent TKA from February 2018 to July 2020 in the Department of Orthopedics, the Second Affiliated Hospital of Fujian Medical University were retrospectively analyzed. According to the inclusion and exclusion criteria, 94 patients (107 knees) were finally included after excluding unqualified cases.

TKA was consistently performed in all patients following the same guidelines and criteria. All surgeries were performed by a dedicated orthopedic diagnosis and treatment team, with a chief surgeon who had over 30 years of orthopedic experience. For patients with PFM, a good patellofemoral trajectory was achieved through adjustment in prosthesis positioning, patellaplasty, and releasing tension in lateral soft tissues. For patients with poor patellofemoral trajectory, the authors used partial resection of the lateral side of the patella, used a swinging saw to shape the patella into a dome prosthesis-like shape, and combined with lateral release of the lateral patellar support band. The problem of patella tilt and patella protrusion can be corrected. The movement track of patella on the femoral trochlea was observed by flexion and extension of the knee during the operation. If the patella had no beating or tilt greater than 15 degrees, it was considered that the movement track of patella was good.

Demographic data, including age, gender, body mass index (BMI), duration of arthritis, and Wiberg patellar type, were collected. Anteroposterior, lateral, and femoral trochlear knee joint weight-bearing assessments were routinely performed both before and after the surgery. Follow-up evaluations were conducted at 3 days, 3 months, and 1 year post-surgery. The Knee Hospital for Special Surgery Knee-Rating Scale (HSS) scores and patellar morphological parameters from radiographs (X-rays) were collected to assess knee joint function. For the analysis, X-ray data before the surgery and 3 months after the surgery were selected, including standardized weight-bearing anteroposterior, lateral, and axial (30 degrees flexion) X-rays. 12

### Outcomes and Measurements

The primary focus of this study was the evaluation of the HSS score of patients 1 year after surgery. Additionally, the study aimed to assess several radiographic parameters (X-rays) (including postoperative patellar displacement, patellar tilt angle, femorotibial angle, valgus angle of the distal femur, valgus angle of the proximal tibia, Insall-Salvati ratio, and thickness of the patella) and observe any complications that might arise postoperatively, such as joint instability, joint stiffness, venous embolism of the lower extremities, and anterior knee pain.

All X-rays imaging were collected retrospectively, and measurements were performed using Centricity Picture Archiving and Communication Systems (PACS) 3.0. The patellar tilt angle and patellar displacement were measured using the axial position as a reference, specifically from the anterior intercondylar line. The patellar tilt angle was calculated based on the patellar transverse axis (angular–angular line) connecting the medial and lateral corners, as described by Grelsamer et al. 33 Both parameters were measured separately by two researchers, and the average of their results was used.

**Fig. 1:**
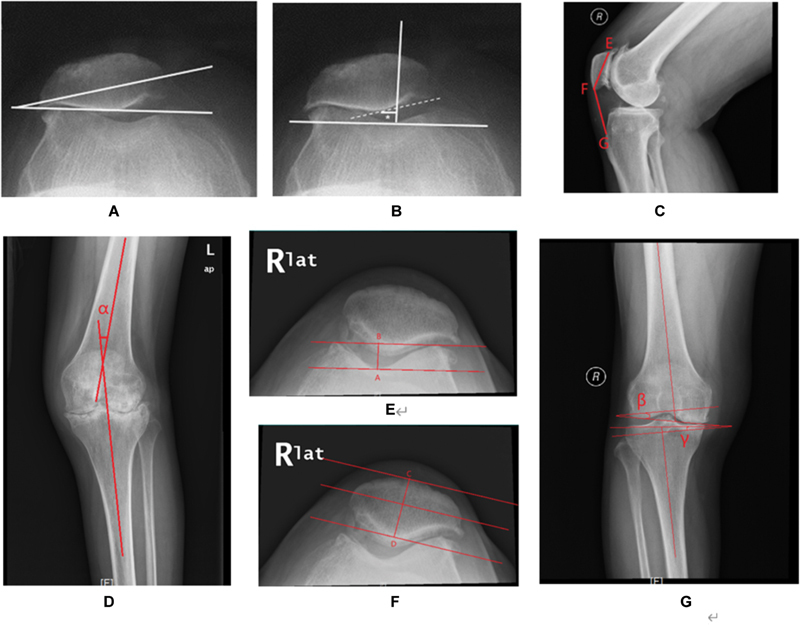
Various measurements and angles related to the patella. ( **A** ) The angle between the patella's transverse axis and its intercondylar line, or anterior patella, is called the patella tilt. It is considered positive when the patella tilts laterally. ( **B** ) Patella displacement refers to the movement of the median crest of the patella from medial to the lateral side. ( **C** ) The Insall-Salvati ratio is the ratio of the patellar tendon length to the patella's longest diagonal (FG/EF). This ratio indicates the vertical height of the patella. ( **D, E** ) The intercondylar sulcus, also known as point A, is the deepest point of the femoral trochlea or the lowest part of the anterior intercondylar line. The distance AB represents the depth of the trochlea. ( **F** ) CD distance refers to the thickness of the anterior and posterior parts of the patella. It is measured from the superficial bone to the median crest of the patella and is perpendicular to the transverse axis of the patella. ( **G** ) The valgus angle of the distal femur (β) and the valgus angle of the proximal tibia (γ).

The angle between the transverse axis of the patella and its anterior intercondylar line was called the patella tilt angle. When the patella tilted laterally, it was considered positive ( Fig. 1A ). Patella displacement referred to the movement of the median crest of the patella from medial to lateral. The point where the median crest was furthest from the transverse axis of the patella was considered the deepest point. As the transverse axis moved downward, the lowest point touched by it was defined as the median crest. If the median crest was lateral to the sulcus, the patella displacement was positive; if it was medial to the sulcus, it was negative ( Fig. 1B ).

The length of the tendon was measured on lateral X-rays from the tibial tubercle to the inferior pole of the patella. The Insall-Salvati ratio was calculated by dividing the patellar length by its maximum diagonal length. This ratio represents the patella's vertical height and was calculated before and after surgery. Normal values for this ratio range from 0.8 to 1.2 ( Fig. 1C ).

To measure the femorotibial angle, the angle formed between the femur's longitudinal and tibial axes was used ( Fig. 1D ). The patella's transverse axis was determined by the line that spans the medial and lateral limits of the articular surface. The intercondylar sulcus, which is the deepest point in the femoral trochlea, was located by referencing the lowest trochlea on the anterior intercondylar line. To reduce observer variability in positioning, the digitally drawn anterior condylar line was adjusted backward until it touched the intercondylar region, where the intercondylar sulcus was the deepest point ( Fig. 1E ).

The measurement of the anterior and posterior thickness of the patella was determined. On lateral X-rays, the distance from the outer surface of the bone to the median crest of the patella was measured perpendicular to the transverse axis of the patella ( Fig. 1F ). The valgus angle of the distal femur and the valgus angle of the proximal tibia were calculated by measuring the inclination of their respective articular surfaces to their backbones ( Fig. 1G ).

The HSS score was used to assess the changes in knee function. The score for each patient was calculated by adding up the scores for each item. Scores over 85 points suggested excellent knee function, scores between 70 and 84 points suggested good knee function, scores between 60 and 69 points suggested fair knee function, and scores below 60 points suggested poor knee function.

### Statistical Analysis

All statistical analyses were conducted using SPSS 25 (IBM Corp., Armonk, NY, USA). Continuous variables following a normal distribution were presented as means ± standard deviations (SD). To compare different groups, we used Student's t-test, and for comparisons within the same group, we employed the paired t-test. Continuous variables with skewed distributions were expressed as medians (ranges). The Mann-Whitney U-test was used to compare the two groups, while Wilcoxon's rank test was used for comparison before and after the surgery. Categorical variables were presented as *n* (frequency) and assessed using the chi-square test. A two-sided *p* < 0.05 was considered statistically significant.

## Results

The study included 94 patients, totaling 107 knees, who underwent TKA. Among them, 37 knees had PFM and 70 knees had PFA. The age, gender, and BMI of the patients with PFM and PFA were similar, as were the preoperative HSS scores between knees with PFM and PFA (all *p* > 0.05) ( Table 1 ). However, there were significant differences in the distribution of patellar Wiberg classification between the knees with PFM and PFA ( *p* < 0.01). This difference in classification might be associated with the presence of PFM.

**Table 1:** Demographic information and clinical characteristics of patients

Variables	PFA (knee = 70)	PFM (knee = 37)	*P* -value
Gender			>0.999
Male	7 (10%)	4 (10.81%)	
Female	63 (90%)	33 (89.19%)	
Height (cm)	161.91 ± 15.89	163.82 ± 16.40	0.560
Weight (kg)	61.22 ± 11.31	59.18 ± 14.49	0.459
BMI (kg/m ^2^ )	22.53 ± 2.72	22.10 ± 3.11	0.461
< 24, *n* (%)	49 (70%)	25 (67.57%)	
≥24, *n* (%)	21 (30%)	12 (32.43%)	
Arthritis course, year	9.29 ± 4.21	10.52 ± 3.08	0.088
Type of patella, *n* (%)			<0.001
Type I	10 (14.29%)	0 (0%)	
Type II	58 (82.86%)	12 (32.43%)	
Type III	2 (2.86%)	16 (43.24%)	
Type IV	0 (0%)	9 (24.32%)	
Valgus–varus deformity,	*n*		
(%)			0.991
Varus	56 (80%)	30 (81.08%)	
Valgus	4 (5.71%)	2 (5.41%)	
Normal	10 (14.29%)	5 (13.51%)	

Abbreviations: BMI, body mass index; PFA, normal patellofemoral alignment; PFM, patellofemoral malalignment.

Notes: Arthritis course: Total time with osteoarthritis.

Varus alignment: MPTA ≤87; valgus alignment: MPTA ≥90.

The imaging parameters measured before surgery, such as patellar displacement and patellar tilt, were found to be associated with PFM. The patellar outward displacement in knees with preoperative PFM changed from 7.01 ± 3.91 mm before surgery to −0.31 ± 2.86 mm after surgery ( *p* < 0.001), and the lateral patellar tilt angle changed from 9.45 ± 7.47 degrees to 6.06 ± 3.61 degrees ( *P* = 0.009). Alignment of the coronal face was evaluated using the femur–tibial axis, distal femur valgus angle, and proximal tibia valgus angle. Regardless of whether PFM was present, the valgus angles of the distal femur (PFA: 9.35 ± 3.27 vs. 6.06 ± 2.51; PFM: 10.23 ± 4.82 vs. 5.87 ± 1.69) and proximal tibia (PFA: 4.20 ± 2.63 vs. 1.36 ± 2.08; PFM: 4.54 ± 2.67 vs. 1.33 ± 1.77) were both lower after TKA compared to before surgery (all *p* < 0.001). However, there were no significant differences in these postoperative radiographic parameters between knees with PFM and PFA ( Table 2 ).

**Table 2:** Preoperative and postoperative patellar morphological parameters from radiographs (X-rays)

Parameters	PFA (knee = 70)	PFM (knee = 37)	*P* -value
Preoperative			
Femorotibial angle (°)	4.40 ± 3.97	5.33 ± 3.84	0.246
Patella tilt angle (°)	4.96 ± 4.48	9.45 ± 7.47	0.001
Patella outward displacement (mm)	0.90 ± 2.06	7.01 ± 3.91	<0.001
Valgus angle of distal femur (°)	9.35 ± 3.27	10.23 ± 4.82	0.265
Valgus angle of proximal tibia (°)	4.20 ± 2.63	4.54 ± 2.67	0.533
Insall-Salvati ratio	1.02 ± 0.11	1.01 ± 0.19	0.769
Thickness of patella (mm)	23.12 ± 1.51	23.18 ± 2.11	0.879
Postoperative			
Femorotibial angle (°)	4.99 ± 2.97**	5.44 ± 1.90	0.344
Patella tilt angle (°)	6.05 ± 4.28	6.06 ± 3.61**	0.992
Patella outward displacement (mm)	0.39 ± 2.75	−0.31 ± 2.86***	0.214
Valgus angle of distal femur (°)	6.06 ± 2.51***	5.87 ± 1.69***	0.689
Valgus angle of proximal tibia (°)	1.36 ± 2.08***	1.33 ± 1.77***	0.941
Insall-Salvati ratio	1.01 ± 0.18	1.02 ± 0.11*	0.723
Thickness of patella (mm)	21.58 ± 1.69	21.51 ± 1.62	0.837

Abbreviations: PFA, normal patellofemoral alignment; PFM, patellofemoral malalignment.

Note: Before *vs.* after operation; * *p* < 0.05, ** *p* < 0.01, *** *p* < 0.001.

Before TKA, there were no significant differences in the HSS scores in each dimension and total scores between the knees with PFM and PFA (all *p* > 0.05). However, after TKA, the HSS scores for knees with preoperative PFM were lower than for PMA (70.35 ± 8.39 vs. 80.47 ± 5.44, *p* < 0.01). The same trend was observed for the postoperative pain score (18.78 ± 5.45 vs. 23.71 ± 4.40, *p* < 0.01) and postoperative function score (14.22 ± 3.23 vs. 19.09 ± 2.60, *p* < 0.01) ( Table 3 ).

**Table 3:** Comparison of HSS scores between the two groups before and after TKA

HSS	PFA (knee = 70)	PFM (knee = 37)	*P* -value
Preoperative			
Pain (score: 30)	8.14 ± 4.44	7.70 ± 4.35	0.624
Function (score: 22)	6.24 ± 3.53	6.32 ± 3.61	0.910
Activity (score: 18)	8.40 ± 3.95	8.43 ± 3.88	0.968
Myodynamia (score: 10)	7.46 ± 2.28	7.57 ± 2.06	0.806
Flexion deformity (score: 10)	4.54 ± 3.53	4.97 ± 3.32	0.542
Instability (score: 10)	5.04 ± 3.49	5.30 ± 3.65	0.725
Total (score: 100)	34.51 ± 8.42	34.73 ± 9.25	0.903
Postoperative			
Pain (score: 30)	23.71 ± 4.40**	18.78 ± 5.45**	<0.001
Function (score: 22)	19.09 ± 2.60**	14.22 ± 3.23**	<0.001
Activity (score: 18)	16.20 ± 1.23**	16.59 ± 1.38**	0.135
Myodynamia (score: 10)	7.89 ± 2.07**	8.22 ± 1.55*	0.395
Flexion deformity (score: 10)	7.31 ± 1.71**	7.49 ± 1.56**	0.610
Instability (score: 10)	8.36 ± 1.60**	7.76 ± 2.01**	0.094
Total (score: 100)	80.47 ± 5.44**	70.35 ± 8.39**	<0.001

Abbreviations: HSS, Hospital for Special Surgery Knee-Rating Scale; PFA, normal patellofemoral alignment; PFM, patellofemoral malalignment; TKA, total knee arthroplasty.

Note: Before *vs.* after operation; * *p* < 0.05, ** *p* < 0.01, *** *p* < 0.001.

The complications recorded after TKA included joint instability, joint stiffness, lower extremity artery embolism, and anterior knee pain. In the PFA group, there were 10 knees with postoperative anterior knee pain (14.29%), whereas in the PFM group there were 13 knees (35.14%) experiencing the same ( *P* = 0.01). There were no significant differences in other complications between the knees with PFM and PFA (all *p* > 0.05) ( Table 4 ). According to the Wiberg classification based on the morphology of the patella, patients with type III patella have a high probability of experiencing anterior knee pain after surgery. The incidence of anterior knee pain in type I patella is low in both groups ( Table 5 ).

**Table 4:** Analysis of postoperative complications of the two groups

Complications	PFA (knee = 70)	PFM (knee = 37)	*P-* value
Joint instability	1 (1.43%)	3 (8.11%)	0.231
Ankylosis	1 (1.43%)	1 (2.70%)	>0.999
Deep vein thrombosis	3 (4.29%)	1 (2.70%)	>0.999
Anterior knee pain	10 (14.29%)	13 (35.14%)	0.013

Abbreviations: PFA, normal patellofemoral alignment; PFM, patellofemoral malalignment.

**Table 5:** Relationship between postoperative anterior knee pain and patella morphological Wiberg classification

Wiberg classification	PFA ( *N* = 10)	PFM ( *N* = 13)	*P-* value
Type I	1 (10%)	0 (0%)	0.708
Type II	7 (70%)	1 (7.69%)	0.03
Type III	2 (20%)	4 (30.77%)	0.036
Type IV	0 (0%)	8 (61.54%)	0.000

Abbreviations: PFA, normal patellofemoral alignment; PFM, patellofemoral malalignment.

## Discussion

This study investigated the impact of preoperative PFM on the prognosis of patients who underwent TKA. The findings revealed that the knees with PFM had lower postoperative HSS scores compared to those with PFA. Moreover, preoperative PFM was potentially linked to experiencing anterior knee pain after TKA.

The HSS scores are used to represent the knee function after TKA and the patient's satisfaction with the outcomes. 11 14 In this study, the PFM group had lower postoperative HSS scores compared to the PFA group, indicating poorer knee function and lower satisfaction in patients with PFM. However, the association between postoperative malalignment and patient-reported outcome measurements (including the HSS) has been inconsistent a previous study. 15 Preoperative PFM has been rarely studied. A study suggested that preoperative mild-to-moderate patellofemoral degeneration or abnormal patellar tilt or congruence should not be a contraindication to TKA, as no correlation was observed with the Knee Injury and Osteoarthritis Outcome Score. 16 Another study found that excessive femoral torsion was not associated with pain after cemented TKA based on the Kujala and patellofemoral score. 17 Therefore, future studies should examine the association between preoperative parameters and postoperative outcomes using multiple scoring systems.

Anterior knee pain, venous thrombosis of the lower extremities, joint stiffness, loosening of the prosthesis, and infection are serious complications that can arise from TKA. 18 In fact, infection rates range from 0.4 to 2% for primary TKA procedures and from 3 to 5% for revision TKA procedures. 19 20 Hinged knees with primary or revision TKA have a high rate of complications. 21 Deep vein thrombosis, particularly after lower extremity joint replacement, is a particularly severe complication that can lead to pulmonary embolism and even death. 22 Furthermore, other complications can also impact patient satisfaction following surgery. 23 24 For this study, patients with postoperative knee infections were excluded, and the most common postoperative complication observed was anterior knee pain. Patients with PFM experienced higher levels of postoperative anterior knee pain compared to those with PFA.

Two patients, with PFM in three knees, were readmitted for a secondary surgery because the patients experienced severe pain in the anterior of their knees. After patellar replacement and lateral soft tissue release, both patients obtained good patellar tracking and significant pain relief. Some past studies have shown that patellar replacement in PFM KOA patients can reduce the incidence of postoperative knee pain, but there are also studies which have shown that patellar replacement does not affect the prognosis of postoperative patients with TKA, so the issue of patellar replacement in PFM KOA patients has always been a continuing debate. Preserving the subchondral bone of the patella is feasible for patellofemoral joint arthroplasty, which can reduce the incidence of patellofemoral joint-related complications, such as patellar wear, fracture, dislocation and subluxation, aseptic loosening, and necrosis. 18 In this study, patients with obvious anterior knee pain in the PFM group could obtain good patellofemoral joint trajectory and significant relief of anterior knee pain after patellar replacement, but other patients with anterior knee pain could still be relieved to some extent by conservative treatment. In the authors' opinion, it remains to be discussed whether patients with PFM need one-stage patellar replacement. Therefore, we still advocate that patients with PFM cannot have patellar replacement first, and there is a chance patients may need a second operation when anterior knee pain occurs. Our future study direction is how to reduce the probability of knee pain in PFM KOA patients after TKA surgery, by improving the operation method, for example, or by further verifying the issue of whether patella replacement can reduce the incidence of knee pain, etc. Primary TKA has been shown to significantly reduce the likelihood of postoperative anterior knee pain in patients with KOA and PFM. 25 However, some studies suggest that TKA does not significantly improve the prognosis for TKA. 26 Regaining the patella during TKA can help prevent complications in the patellofemoral joint, such as patellar wear, fractures, dislocations, subluxations, aseptic loosening, and necrosis. 27 However, the use of femoral prosthesis can alter the original patellofemoral trajectory and biomechanics of the patella. 28 Keeping the patella can lead to negative outcomes because the prolonged contact between the cartilage and metal can result in cartilage damage. 29 Patients in the PFM group who experience anterior knee pain can find relief and improve their patellofemoral trajectory after undergoing TKA; conservative treatment may also provide some improvement. The timing of patella replacement in patients with PFM is a subject of debate, as it is uncertain whether one-stage TKA is necessary for all patients. Patients with anterior knee pain have the option of secondary surgery to address this issue. Future research should aim to lower the risk of anterior knee pain in KOA patients with PFM who have undergone TKA. This could involve refining surgical technique or further exploring the necessity of one-stage TKA.

When evaluating young patients with anterior knee pain and patellofemoral instability, axial X-rays of the patellofemoral joint are commonly used to assess the coronal relationship between the autologous patella and the trochlea. This relationship is considered important. 30 Although axial X-rays are not typically necessary for evaluating patients undergoing TKA, they can help predict the likelihood of intraoperative trajectory malalignment. 31 Compared to other methods like computed tomography (CT) scan and magnetic resonance imaging (MRI), 32 axial X-rays of patellar trajectories are easier to perform. This study demonstrates that the results of these X-rays are highly consistent and can provide valuable information for surgical planning in patients undergoing TKA. 33 Understanding patellofemoral mechanics before surgery can lead to more targeted and accurate preoperative planning, as well as better outcomes and reduced pain after surgery. 34 Therefore, it is recommended that clinicians perform a patellar trajectory analysis before TKA to improve surgical planning and patient prognosis, especially for patients with knee osteoarthritis who experience anterior knee pain and have issues with patellofemoral mechanics.

This study has several limitations. First, it only explored the relationship between recovery after TKA and patellar trajectory in patients with KOA at a single center with a small sample size. This limited scope could introduce selection bias and local management bias. Second, the long follow-up period and the use of telephone follow-up made it challenging to categorize complications as occurring soon after discharge or later in the 12-month follow-up period. This may have led to an overestimation of surgery-related complications. Third, the study did not conduct subgroups analysis to investigate the reasons for differences in HSS scores between the two groups. Fourth, the study lacks a control group for assessing the effects of PFA on postoperative outcomes, as well as randomization, which may introduce confounding variables and affect the validity of the findings. Consequently, further research is required to validate the findings of this study.

In conclusion, our results showed that PFM was significantly associated with postoperative anterior knee pain, and the postoperative HSS score of patients with PFM was lower than that of patients with normal trajectory. PFM affected the functional recovery and prognosis of patients with osteoarthritis after TKA. Therefore, the analysis of patellar trajectory should be performed before operation to better guide clinicians in making surgical plans to improve the prognosis of patients. However, we suspect that the reason why patients in the PFM group are more likely to have anterior knee pain after surgery may be due to the imbalance of muscles and soft tissues after PFM correction, and the specific mechanism needs to be further studied and verified.
